# Sudden Cardiac Death: The Most Feared but Potentially Preventable Presentation of Wolff-Parkinson-White Syndrome

**DOI:** 10.1155/2021/9083144

**Published:** 2021-11-18

**Authors:** Ana Rita Pereira, Alexandra Briosa, Rita Miranda, Sofia Sequeira Almeida, Luís Brandão, Hélder Pereira

**Affiliations:** Cardiology Department, Hospital Garcia de Orta, Avenida Torrado da Silva, 2805-267 Almada, Portugal

## Abstract

*Background*. Wolff-Parkinson-White syndrome is an uncommon cardiac disorder characterized by the presence of one or more accessory pathways that predispose patients to frequent episodes of arrhythmias. The prognosis is usually good, but there is a lifetime risk of malignant arrhythmias and sudden cardiac death. *Case Summary*. A 25-year-old male presented a witnessed out-of-hospital cardiac arrest with ventricular fibrillation rhythm. Due to rapid initiation of prehospital advanced life support, return of spontaneous circulation was observed. During the transport to the hospital, an irregular wide complex tachycardia suggestive of preexcited atrial fibrillation with haemodynamic instability was also observed and a synchronized shock was applied. Baseline 12-lead electrocardiogram was compatible with sinus rhythm and ventricular preexcitation pattern. After clinical stabilization, an electrophysiological study was performed confirming the presence of a left anterolateral accessory pathway with a short antegrade effective refractory period. Successful radiofrequency catheter ablation was achieved. *Discussion*. The reported clinical case recalls fundamental features of the Wolff-Parkinson-White syndrome and outlines the increasing evidence and importance of the invasive risk stratification and even catheter ablation in asymptomatic patients who suffer from this uncommon disease that may have a dramatic and fatal initial clinical manifestation.

## 1. Introduction

Wolff-Parkinson-White (WPW) syndrome is an uncommon cardiac disorder characterized by the presence of one or more accessory pathways (APs) that predispose patients to arrhythmias [1–3]. The prognosis is usually good, but there is a lifetime risk of malignant arrhythmias and sudden cardiac death (SCD), which may be the first presentation of the disease [4]. The management of symptomatic patients is well established in the international guidelines for a long time [2, 3]. Otherwise, the approach of asymptomatic preexcitation patients is less defined. Herein, we present a clinical case that intends to resemble the potential lethality of the syndrome and to emphasize the importance of risk stratification of asymptomatic patients.

## 2. Case Presentation

**Figure 1:**
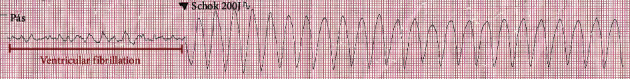
Twelve-lead electrocardiogram showing ventricular fibrillation. A biphasic shock of 200 J was applied with conversion to a regular wide complex tachycardia with a heart rate of 215 bpm.

**Figure 2:**
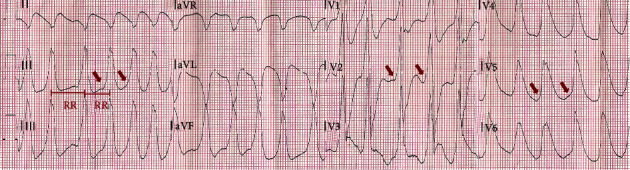
Twelve-lead electrocardiogram showing atrial fibrillation with a rapid preexcited ventricular response. Irregular RR intervals, wide QRS complexes with varying QRS width, and initial delta wave (arrow). The shortest preexcited RR interval is nearly 300 ms.

A 25-year-old male with no personal or familial history of cardiovascular disease presented a witnessed out-of-hospital cardiac arrest. Prehospital emergency service was immediately activated, and cardiopulmonary resuscitation was promptly started. When the prehospital critical care team arrived, the cardiac rhythm was ventricular fibrillation (VF) and a biphasic shock of 200 J was applied with conversion to a regular wide complex tachycardia with a heart rate of 215 bpm (Figure 1). Advanced life support was proceeded. A total of four defibrillations was applied until return of spontaneous circulation. During the transport to the hospital, the patient became unstable again presenting irregular wide complex tachycardia suggestive of preexcited atrial fibrillation (AF) and a synchronized shock was applied (Figure 2). On arrival at the hospital, the patient was haemodynamically stable, with no significant changes on cardiopulmonary examination.

## 3. Investigation

**Figure 3:**
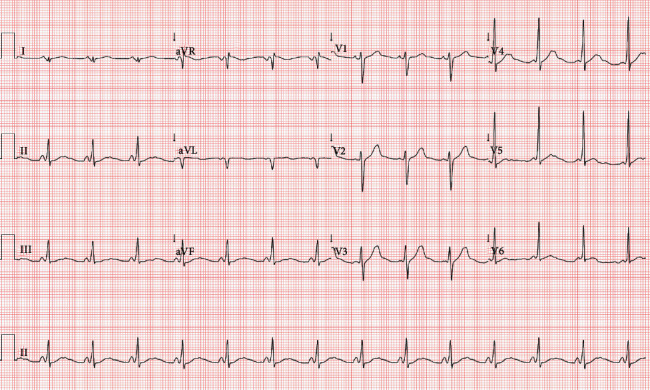
Twelve-lead electrocardiogram after conversion to sinus rhythm (=baseline electrocardiogram). Shortened PR interval, delta wave, and secondary repolarization abnormalities are seen. This ECG is indicative of WPW syndrome, and a left anterolateral AP is suggested by negative QRS in V1 lead, QS complex in aVL lead, and positive delta wave in V3-V6 leads and inferior leads.

No medication or drug abuse was reported. Serum potassium and magnesium levels were normal, and transthoracic echocardiogram excluded structural heart disease. However, 12-lead electrocardiogram (ECG) was compatible with sinus rhythm and ventricular preexcitation with shortened PR interval, delta waves in V3-V6 and inferior leads, and secondary ventricular repolarization abnormalities (Figure 3).

## 4. Diagnosis and Treatment

**Figure 4:**
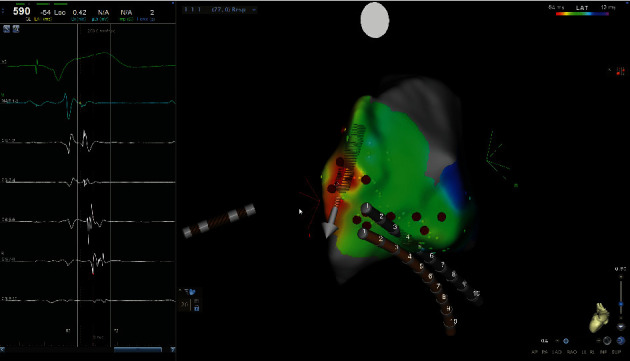
3D mapping and intracavitary recordings confirming the presence of a left anterolateral AP.

An electrophysiological study (EPS) was performed. Two catheters were positioned via the right femoral vein: an Inquiry decapolar 6F catheter (Abbott, St Paul, MN, USA) in the coronary sinus and a quadripolar catheter CRD 6F (St Jude Medical Inc, St Paul, MN, USA) in the right ventricle for His activity tracing and ventricular stimulation. Fluoroscopy integrated 3D-mapping using the CARTO 3 system (Biosense-Webster, Diamond Bar, CA, USA), and intracavitary recordings confirmed the presence of ventricular preexcitation via a left anterolateral AP (Figure 4) with a short antegrade effective refractory period (AERP) of 210 ms, anterograde block cycle length of 260 ms, and retrograde block cycle length of 250 ms. During the procedure, an orthodromic atrioventricular reentrant tachycardia (AVRT) was induced with a cycle length of 410 ms. Left heart cavities were approached using a fast-cath transseptal guiding introducer SL1 curve 8.5F (St Jude Medical Inc, St Paul, MN, USA) and a BRK transseptal needle (Abbott, St Paul, MN, USA). Radiofrequency ablation was performed using a ThermoCool SmartTouch DF curve catheter (Biosense-Webster, Diamond Bar, CA, USA). Conduction over the AP was successfully interrupted within 1 second of energy delivery (35 W). At the end of the procedure, there was no evidence of ventricular preexcitation. No periprocedural complications ensued.

## 5. Outcome and Follow-Up

**Figure 5:**
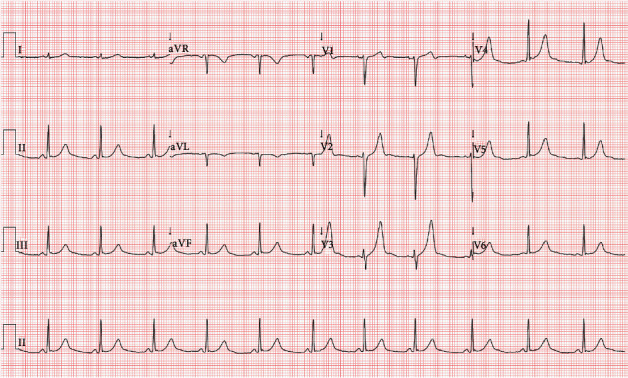
Twelve-lead electrocardiogram after catheter ablation. No delta waves and PR interval at the lower limit of normal but revealed peaked upright T waves in leads V3-V6 and inferior leads.

The patient was discharged three days after the procedure. At discharge, 12-lead ECG showed sinus rhythm and absence of preexcitation pattern with no delta waves and PR interval at the lower limit of normal (PR 120 ms) but revealed peaked upright T waves in leads V3-V6 and inferior leads (Figure 5). At the 6-month follow-up, the patient was asymptomatic, no tachyarrhythmias were documented, and 12-lead ECG had no change relating to that at hospital discharge.

## 6. Discussion

WPW pattern occurs in 0.1 to 0.2% of the general population [5, 6]. The proportion of patients with WPW who are truly asymptomatic is unknown. Nonetheless, there seems to be agreement within the literature that more than 90% of children, approximately 65% of adolescents, and 40% of patients older than 30 years of age are asymptomatic [7, 8]. A rough extrapolation of the results published by Munger et al. reveals that an asymptomatic child or adolescent accumulates a 45% probability of remaining asymptomatic, with no arrhythmia, throughout their lifetime [7–9].

At the adult age, symptoms generally onset at a mean of 28 years [9]. At paediatric age, a bimodal distribution is observed with a first peak in the first month of life (including prenatally) and a secondary, more diffuse peak through the school age years [5]. Symptoms are mainly related to the occurrence of tachyarrhythmias: AVRT in 80% of the cases and AF in 20 to 30%, which is usually triggered by AVRT in the presence of high atrial vulnerability [1, 4, 7, 8]. Preexcited AF is a potentially life-threatening arrhythmia since rapid conduction to the ventricle over an AP with a short AERP may degenerate into VF and consequently cardiac arrest, as observed in the presented clinical case [1, 4]. In fact, SCD is the most feared clinical manifestation, occurring generally around the age of 20 to 30 years, as seen in our patient [7, 8, 10–12]. A 10-year risk of SCD of 0.15-0.24% [9] and an annual incidence of 0.01-0.5% in adults [9, 13] and 0.001-0.2% in children [13, 14] are estimated. Alarmingly, between 12% and 53% (average of 27%) of WPW patients presenting with cardiac arrest had no previous symptoms or knowledge of their diagnosis [7, 8, 10–12, 15]. Because of the increasing use of ECG in the general population, the number of asymptomatic patients with WPW patterns is expected to increase [5].

For the reasons above, risk assessment and management of asymptomatic preexcitation patients both in paediatric and adult age have been the focus of several recent publications [6, 11, 16–23]. Delise and Sciarra reviewed the results of these studies concluding that asymptomatic WPW is far from rare, absence of symptoms is not in itself a marker of low risk and do not predict the findings of risk stratification, electrophysiologic study has a high predictive value, and prophylactic ablation is highly effective in preventing fatal events [8]. All this new information is also reflected in the 2019 European Society of Cardiology (ESC) guidelines for the management of adult patients with supraventricular tachycardia [1].

Regarding risk assessment, several clinical features, invasive markers, and noninvasive markers are already recognized. Younger age and male gender are associated with an increased risk of SCD [13, 16, 20]. Invasive markers assessed by EPS of high risk comprise inducibility of AP-mediated tachycardia in the baseline state or during isoproterenol infusion [16, 20, 24], multiple APs [20], and demonstration of rapid conduction through the AP to the ventricles such as short preexcited RR interval during AF (≤250 ms) or a short AERP of the AP (≤250 ms) [16, 18, 20]. Abrupt and complete normalization of the PR interval with loss of delta wave during exercise testing or following procainamide, propafenone, or disopyramide administration has been considered a noninvasive marker of low risk [6, 9]. These risk markers are also applicable to the paediatric population since several dedicated studies have already demonstrated the validity of the noninvasive [15, 25, 26] and invasive [15, 17, 19, 27] parameters in this age.

In the 2019 ESC guidelines [1], there was no change in the symptomatic patients' approach but there was an upgrade of the asymptomatic patients' management. Invasive screening with an EPS is now recommended for patients with high-risk occupations or competitive athletes and should be considered for the remaining patients. This makes the level of recommendation for EPS higher than that for noninvasive evaluation in adults' risk stratification. In contrast, an exercise stress test remains a routine part of the evaluation of asymptomatic children, and risk stratification by EPS is only recommended in those who do not have a reassuring pattern of clear loss of preexcitation during noninvasive tests [5].

Concerning management, previous guidelines [2, 3] recommended routine EPS and catheter ablation only in symptomatic patients. In the 2019 ESC guidelines [1], if a high-risk AP is diagnosed, catheter ablation is recommended and may be considered in low-risk preexcitation in appropriately experienced centres. Catheter ablation of an AP, when performed by an experienced operator, is associated with a high cure rate (>95%) and low risk (<0.5%) of major complications [16, 28]. About asymptomatic children, catheter ablation has a class IIb indication in patients 5 years and older and a class 3 indication for patients younger than 5 years since low weight and height are associated with a higher risk of complications [29]. Nevertheless, catheter ablation at paediatric age has also a high success rate (>95%) [14, 30].

Focusing on the presented clinical case, it mainly recalls WPW syndrome as a cause of SCD even in patients without previous symptoms and highlights the importance of risk stratification. The reported patient had several risk features (young age, male gender, inducibility of AVRT during EPS, and short AERP of the AP) that would have recommended AP ablation and avoid the life-threatening event if the WPW pattern had been earlier diagnosed. Nevertheless, the diagnosis of completely asymptomatic patients will remain challenging once a 12-lead ECG as a screening test for detection of cardiovascular disease in healthy and asymptomatic young people has not yet proved cost-effectiveness and therefore is not recommended [31].

An additional learning point is the revision of the different electrocardiographic features of WPW syndrome, namely, sinus rhythm with a preexcitation pattern, AVRT, and preexcited AF. A final teaching point is a discussion about the large, peaked T waves that may appear following ablation with concordant polarity in leads where the delta wave was most noticeable and/or positive. It is called a classic postablation memory T wave pattern, considered evidence of successful ablation [32].

In summary, the reported clinical case recalls fundamental features of the WPW syndrome and outlines the increasing evidence and importance of the invasive risk stratification in asymptomatic patients who suffer from this uncommon disease that may have a dramatic first clinical manifestation.

## Data Availability

Data can be available under request of the editor of the journal.

## References

[1] Brugada J., Katritsis D. G., Arbelo E. (2020). 2019 ESC guidelines for the management of patients with supraventricular tachycardiathe task force for the management of patients with supraventricular tachycardia of the European Society of Cardiology (ESC). *European Heart Journal*.

[2] Blomström-Lundqvist C., Scheinman M. M., Aliot E. M. (2003). ACC/AHA/ESC guidelines for the management of patients with supraventricular arrhythmias∗--executive summary:. *Journal of the American College of Cardiology*.

[3] Page R. L., Joglar J. A., Caldwell M. A. (2016). 2015 ACC/AHA/HRS guideline for the management of adult patients with supraventricular tachycardia: a report of the American College of Cardiology/American Heart Association Task Force on Clinical Practice Guidelines and the Heart Rhythm Society. *Heart Rhythm*.

[4] Fengler B. T., Brady W. J., Plautz C. U. (2007). Atrial fibrillation in the Wolff-Parkinson-White syndrome: ECG recognition and treatment in the ED. *The American Journal of Emergency Medicine*.

[5] Triedman J. K. (2009). Management of asymptomatic Wolff-Parkinson-White syndrome. *Heart*.

[6] Skov M. W., Rasmussen P. V., Ghouse J. (2017). Electrocardiographic preexcitation and risk of cardiovascular morbidity and Mortality. *Circulation. Arrhythmia and Electrophysiology*.

[7] Delise P., Sciarra L. (2007). Asymptomatic Wolff-Parkinson-White: what to do. Extensive ablation or not?. *Journal of Cardiovascular Medicine*.

[8] Delise P., Sciarra L. (2020). Sudden cardiac death in patients with ventricular preexcitation. *Cardiac Electrophysiology Clinics*.

[9] Munger T. M., Packer D. L., Hammill S. C. (1993). A population study of the natural history of Wolff-Parkinson-White syndrome in Olmsted County, Minnesota, 1953-1989. *Circulation*.

[10] Klein G., Bashore T., Sellers T. D., Pritchett E., Smith W., Gallagher J. (1979). Ventricular fibrillation in the Wolff-Parkinson-White syndrome. *The New England Journal of Medicine*.

[11] Torner P., Brugada P., Smeets J. (1991). Ventricular fibrillation in the Wolff-Parkinson-White syndrome. *European Heart Journal*.

[12] Timmermans C., Smeets J., Rodriguez L., Vrouchos G., van den Dool A., Wellens H. (1995). Aborted sudden death in the Wolff-Parkinson-White syndrome. *The American Journal of Cardiology*.

[13] Obeyesekere M. N., Leong-Sit P., Massel D. (2012). Risk of arrhythmia and sudden death in patients with asymptomatic preexcitation: a meta-analysis. *Circulation*.

[14] Pappone C., Manguso F., Santinelli R. (2004). Radiofrequency ablation in children with asymptomatic Wolff–Parkinson–White syndrome. *The New England Journal of Medicine*.

[15] Etheridge S. P., Escudero C. A., Blaufox A. D. (2018). Life-threatening event risk in children with Wolff-Parkinson-White syndrome: a multicenter international study. *JACC: Clinical Electrophysiology*.

[16] Pappone C., Vicedomini G., Manguso F. (2014). Wolff-Parkinson-White syndrome in the era of catheter ablation insights from a registry study of 2169 patients. *Circulation*.

[17] Santinelli V., Radinovic A., Manguso F. (2009). The natural history of asymptomatic ventricular pre-excitation: a long-term prospective follow-up study of 184 asymptomatic children. *Journal of the American College of Cardiology*.

[18] Klein G. J., Prystowsky E. N., Yee R., Sharma A. D., Laupacis A. (1989). Asymptomatic Wolff-Parkinson-White. Should we intervene?. *Circulation*.

[19] Kubuš P., Vít P., Gebauer R. A., Materna O., Janoušek J. (2014). Electrophysiologic profile and results of invasive risk stratification in asymptomatic children and adolescents with the Wolff-Parkinson-White electrocardiographic pattern. *Circulation. Arrhythmia and Electrophysiology*.

[20] Santinelli V., Radinovic A., Manguso F. (2009). Asymptomatic ventricular preexcitation a long-term prospective follow-up study of 293 adult patients. *Circulation. Arrhythmia and Electrophysiology*.

[21] Pappone C., Vicedomini G., Manguso F. (2012). Risk of malignant arrhythmias in initially symptomatic patients with Wolff-Parkinson-White syndrome: results of a prospective long-term electrophysiological follow-up study. *Circulation*.

[22] Bunch T. J., May H. T., Bair T. L. (2015). Long-term natural history of adult Wolff-Parkinson-White syndrome patients treated with and without catheter ablation. *Circulation. Arrhythmia and Electrophysiology*.

[23] Brembilla-Perrot B., Marçon O., Chometon F. (2006). Supraventricular tachyarrhythmia as a cause of sudden cardiac arrest. *Journal of Interventional Cardiac Electrophysiology*.

[24] Leitch J. W., Klein G. J., Yee R., Murdock C. (1991). Prognostic value of electrophysiology testing in asymptomatic patients with Wolff-Parkinson-White pattern. *Circulation*.

[25] Wackel P., Irving C., Webber S., Beerman L., Arora G. (2012). Risk stratification in Wolff-Parkinson-White syndrome: the correlation between noninvasive and invasive testing in pediatric patients. *Pacing and Clinical Electrophysiology*.

[26] Bershader R., Berul C., Cecchin F. (2007). Exercise testing for risk assessment in pediatric Wolff-Parkinson-White syndrome. *Heart Rhythm*.

[27] Fazio G., Mossuto C., Basile I. (2009). Asymptomatic ventricular pre-excitation in children. *Journal of Cardiovascular Medicine*.

[28] Bravo L., Atienza F., Eidelman G. (2018). Safety and efficacy of cryoablation vs. radiofrequency ablation of septal accessory pathways: systematic review of the literature and meta-analyses. *Europace*.

[29] Friedman R. A., Walsh E. P., Silka M. J. (2002). NASPE expert consensus conference: radiofrequency catheter ablation in children with and without congenital heart disease. Report of the writing *committee*. *Pacing and Clinical Electrophysiology*.

[30] Kugler J. D., Danford D. A., Houston K. A., Felix G. (2002). Pediatric radiofrequency catheter ablation registry success, fluoroscopy time, and complication rate for supraventricular tachycardia: comparison of early and recent eras. *Journal of Cardiovascular Electrophysiology*.

[31] Maron B. J., Friedman R. A., Kligfield P. (2014). Assessment of the 12-lead ECG as a screening test for detection of cardiovascular disease in healthy general populations of young people (12-25 years of age). *Circulation*.

[32] Helguera M. E., Pinski S. L., Sterba R., Trohman R. G. (1994). Memory T waves after radiofrequency catheter ablation of accessory atrioventricular connections in Wolff-Parkinson-White syndrome. *Journal of Electrocardiology*.

